# Performance of Machine Learning Models for Predicting Occult Nodal Metastasis in Oral Cavity Squamous Cell Carcinoma: A Systematic Review and Diagnostic Accuracy Meta-Analysis

**DOI:** 10.3390/cancers18142271

**Published:** 2026-07-15

**Authors:** Jonathan M. Hughes, Sammy Y. Gao, Shaun A. Nguyen, Jason G. Newman

**Affiliations:** Department of Otolaryngology–Head and Neck Surgery, Medical University of South Carolina, Charleston, SC 29425, USA

**Keywords:** head and neck cancer, oral cavity, squamous cell carcinoma, occult nodal metastasis, machine learning, elective neck dissection

## Abstract

Background/Objectives: Occult cervical nodal metastasis drives prognosis in oral cavity squamous cell carcinoma (OCSCC), yet current tools for risk stratification in clinically node-negative (cN0) patients are imperfect. Machine learning (ML) models have been proposed to refine selection for elective neck dissection (END), but their diagnostic performance and generalizability are unclear. Methods: We performed a diagnostic test accuracy systematic review and meta-analysis of ML models predicting occult nodal metastasis in adults with cN0 OCSCC. Eligible studies evaluated an ML-based model, used pathologic nodal status as the reference standard, and reported or allowed reconstruction of sensitivity and specificity. Internal and external validation were distinguished; quantitative synthesis was restricted to non-overlapping external validation cohorts. Diagnostic performance was synthesized with a bivariate random-effects hierarchical summary receiver operating characteristic (HSROC) model, with prespecified sensitivity analyses restricting to lower-risk patient-selection cohorts, models using only preoperative predictors, and non-outlying cohorts. Results: Thirteen retrospective studies (4730 patients) met inclusion; pooled occult nodal metastasis prevalence was 23.6% (crude 20.4%). Eight studies reported only internal validation; five provided six external validation cohorts. Across these external cohorts, pooled sensitivity was 0.79 (95% CI, 0.64–0.89) and specificity 0.84 (95% CI, 0.70–0.93). Negative predictive values were consistently high (0.94–0.98), whereas positive predictive values were modest (0.39–0.85). Sensitivity analyses yielded similar summary estimates with wider confidence intervals. Conclusions: Externally validated ML models for predicting occult nodal metastasis in cN0 OCSCC show promise but remain insufficiently validated to guide END in routine practice.

## Simple Summary

Oral cavity cancer can metastasize to lymph nodes in the neck even when those nodes appear normal on examination and imaging. Because this hidden, or occult, nodal disease strongly influences survival, treatment of the neck is often considered. Such treatment carries real risks, however, and many patients ultimately gain no benefit from it. To refine this decision, researchers have developed machine learning models that learn from patient data to predict occult nodal disease, yet whether these tools perform reliably across patient populations remains unclear. In this study, the authors reviewed all such models and combined results from those tested on independent patient groups. They found the tools show promise for safely identifying low-risk patients but are not yet dependable enough for routine use. This work clarifies where these models currently stand and what must still be proven before they can guide neck treatment.

## 1. Introduction

Oral cavity squamous cell carcinoma (OCSCC) carries a significant burden of mortality, with cervical lymph node metastasis representing one of the most important determinants of survival [1,2]. Even among patients presenting with cN0 disease, occult nodal metastasis is identified in over 20% of surgical specimens [3], a rate that has long justified elective management of cervical lymphatics [4]. Yet the decision to perform elective neck dissection (END) in cN0 OCSCC remains a consequential and long-contested choice in head and neck surgical oncology [5,6]. Neck dissection carries meaningful morbidity, including risk of shoulder dysfunction, lymphedema, chyle leak, and nerve injury [7,8,9,10], while observation risks undertreating a disease in which nodal upstaging worsens five-year survival by approximately 50% [8]. Current NCCN guidelines strongly recommend consideration of END when depth of invasion (DOI) exceeds 3 mm [11], but its sensitivity remains imperfect, and a meaningful proportion of patients with invasion below this threshold harbor nodal disease that goes untreated.

The limitations of DOI as a sole discriminator have driven sustained interest in multimodal predictive approaches capable of integrating the full complexity of tumor biology, radiographic anatomy, and patient characteristics into a single risk estimate [8,12,13]. Machine learning (ML) offers a framework suited to this task, and unlike conventional logistic regression, ML models can capture nonlinear relationships and high-dimensional interactions among clinical, pathologic, radiomic features without requiring a priori variable specification [14]. Over the past decade, a growing body of studies has applied ML to the prediction of occult nodal metastasis in OCSCC, reporting models with favorable area under the receiver operating characteristic curve (AUC). These results have generated enthusiasm for ML as a potential decision-support tool to individualize neck management by stratifying patients into those who may safely forego END and those for whom it is imperative [13].

However, enthusiasm must be tempered by methodological scrutiny. Predictive models evaluated only on internal validation datasets are known to overestimate true performance due to optimism from overfitting to the training distribution. The critical and frequently overlooked question is not whether a model performs well on held-out data from its own institution, but whether it generalizes to new patients across different surgical practices, imaging protocols, and pathologic processing standards. External validation, the application of a trained model to an entirely independent cohort, is only the second step to develop a clinically usable model using traditional model development frameworks [15], yet it remains the exception for ML models in oncology literature [16]. Without it, even technically sophisticated models offer limited guidance to the practicing surgeon.

To date, no systematic review has rigorously synthesized the diagnostic performance of ML models for occult nodal metastasis prediction in cN0 OCSCC with specific attention to external validation as an evidentiary threshold for clinical inference. Prior reviews have catalogued model characteristics broadly but have pooled internal and external performance estimates without distinction, potentially overstating the true state of the evidence. The present study addresses this gap through a systematic review and diagnostic accuracy meta-analysis restricted to externally validated models, employing a hierarchical summary receiver operating characteristic framework to derive pooled estimates of sensitivity and specificity that reflect genuine out-of-sample performance. By anchoring conclusions to external validation alone, this review aims to provide surgeons, tumor boards, and health technology assessors with an honest appraisal of where ML currently stands, and what remains required before these tools can meaningfully inform the decision to perform END in oral cavity cancer.

## 2. Materials and Methods

### 2.1. Search Strategy

This study was conducted according to the Preferred Reporting Items for Systematic Reviews and Meta-Analyses (PRISMA) guidelines [17]. A comprehensive literature search was conducted of the Cochrane Library (Wiley), PubMed (National Library of Medicine—National Institutes of Health), CINAHL Complete (EBSCOhost), and Scopus (Elsevier) databases from inception through 1 December 2025. Search terms (Table S3) included a combination of free-text keywords, MeSH terms, and Boolean operators across three conceptual domains: OCSCC, ML methodologies, and occult nodal metastasis. All articles were imported into Covidence review management software (Veritas Health Innovation Ltd., Melbourne, Australia, https://www.covidence.org/, accessed on 1 December 2025). Prospective registration was attempted via PROSPERO, but diagnostic test accuracy (DTA) reviews are not accepted on that platform, and the review was not deposited in an alternative registry (PROSPERO, https://www.crd.york.ac.uk/PROSPERO/help/eligibility, accessed on 1 December 2025). The review was conducted and reported in accordance with the PRISMA-DTA guideline.

### 2.2. Selection Criteria and Data Extraction

Studies were eligible for inclusion if they met all of the following criteria: (1) the study population comprised adult patients with cN0 OCSCC; (2) the index test was a ML prediction model developed to predict occult cervical lymph node metastasis; (3) the reference standard was pathologic nodal staging from neck dissection specimen; and (4) the study reported sufficient performance data: sensitivity, specificity, AUC, or contingency table values to permit extraction or reconstruction of diagnostic accuracy estimates. No restriction was placed on clinical T-stage, model architecture, input feature domain, or training dataset source. Both retrospective and prospective study designs were eligible. Studies were excluded if they applied conventional logistic regression without a ML component, included subsites beyond the oral cavity without subgroup data permitting isolation of oral cavity cases, used a reference standard other than pathologic nodal assessment, or reported performance metrics insufficient for contingency table reconstruction. For the diagnostic accuracy meta-analysis, an additional eligibility criterion was applied: only studies reporting non-overlapping external validation cohorts were included in pooled analysis, defined as validation patient populations in which no individual appeared in the training dataset. Institutional co-occurrence between training and validation sources was not itself an exclusion criterion, as multi-institutional training datasets routinely encompass the same centers contributing to subsequent validation efforts; what was required was that no individual patient appeared in both datasets. Studies reporting external validation on cohorts with documented overlap with the training dataset were assessed descriptively but excluded from HSROC synthesis.

### 2.3. Risk of Bias and Study Quality Assessment 

Risk of bias was assessed for each included study using the Quality Assessment of Diagnostic Accuracy Studies-2 (QUADAS-2) tool (University of Bristol, Bristol, UK), which evaluates four domains: patient selection, index test conduct and interpretation, reference standard conduct and interpretation, and flow and timing. Two reviewers (J.M.H. and S.Y.G.) independently assessed each domain and rated bias as low, unclear, or high. Disagreements were resolved by a third reviewer (S.A.N.). Concerns regarding applicability were assessed separately for the patient selection, index test, and reference standard domains. For studies contributing cohorts to the HSROC meta-analysis, domain-level bias ratings were considered when interpreting pooled estimates.

### 2.4. Data Analysis

**Figure 1:**
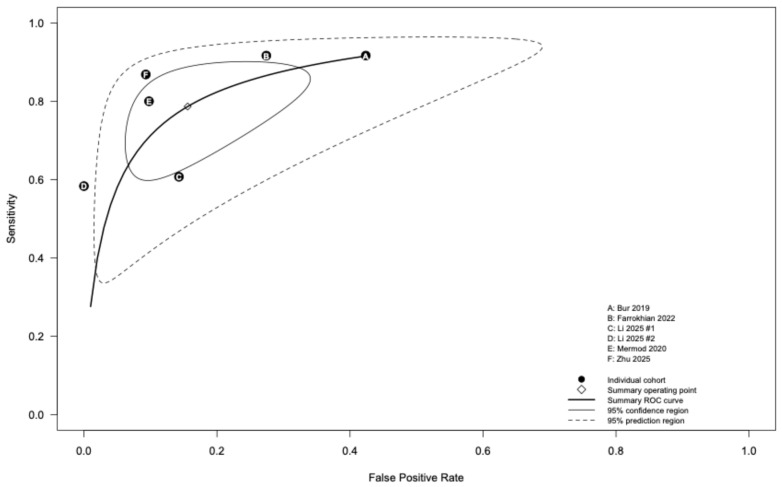
HSROC plot of the six external validation cohorts. Summary operating point (sensitivity 0.79, specificity 0.84); solid ellipse, 95% confidence region; dashed ellipse, 95% prediction region; solid line, HSROC curve from the bivariate model over the observed data range [13,19,20,21,22].

A diagnostic accuracy meta-analysis was conducted including only non-overlapping external validation cohorts, defined as validation datasets in which no individual patient appeared in the training set, irrespective of institutional overlap. Contingency tables (true positives, false positives, false negatives, and true negatives) were reconstructed from each study’s reported sensitivity, specificity, and outcome prevalence, extracted to two decimal places to match the precision of the original reports. From these values and each study’s reported sample size, 2 × 2 contingency-table cell counts were reconstructed as integers; these integer counts, not the derived proportions, were the inputs to the bivariate model (Supplementary Table S1). Diagnostic performance was synthesized using a bivariate random-effects model within a HSROC framework, which jointly models sensitivity and specificity while accounting for between-study heterogeneity and threshold effects. Pooled estimates of sensitivity and specificity with 95% confidence intervals (CI) were derived, and HSROC curves were generated. The bivariate model was prespecified as the recommended approach for DTA synthesis and fitted with the reitsma function in the mada package using restricted maximum likelihood [18]. The bivariate model was fit by restricted maximum likelihood with the sensitivity–specificity correlation estimated rather than fixed. With only six external cohorts this parameter is weakly identified; the summary operating point, not the estimated correlation, is the intended object of interpretation. The prediction region in Figure 1 is reported alongside the confidence region to reflect residual between-study variability. Prespecified sensitivity analyses repeated the HSROC modeling after restricting to external cohorts from studies at lower risk of bias in the QUADAS-2 patient selection domain, restricting to models based solely on preoperative clinical and/or radiomic predictors, and excluding outlying cohorts identified on the HSROC plot. Because only six external cohorts were available, the variance and covariance parameters are weakly identified, so the pooled synthesis is interpreted as exploratory and read alongside the individual cohort estimates. The same specification was applied in the sensitivity analyses restricted to three or four cohorts, where the results are interpreted descriptively. A univariate random-effects cross-check of sensitivity and specificity is reported in Supplementary Table S2. All analyses were performed in R (version 4.5.2).

## 3. Results

### 3.1. Literature Search

**Figure 2:**
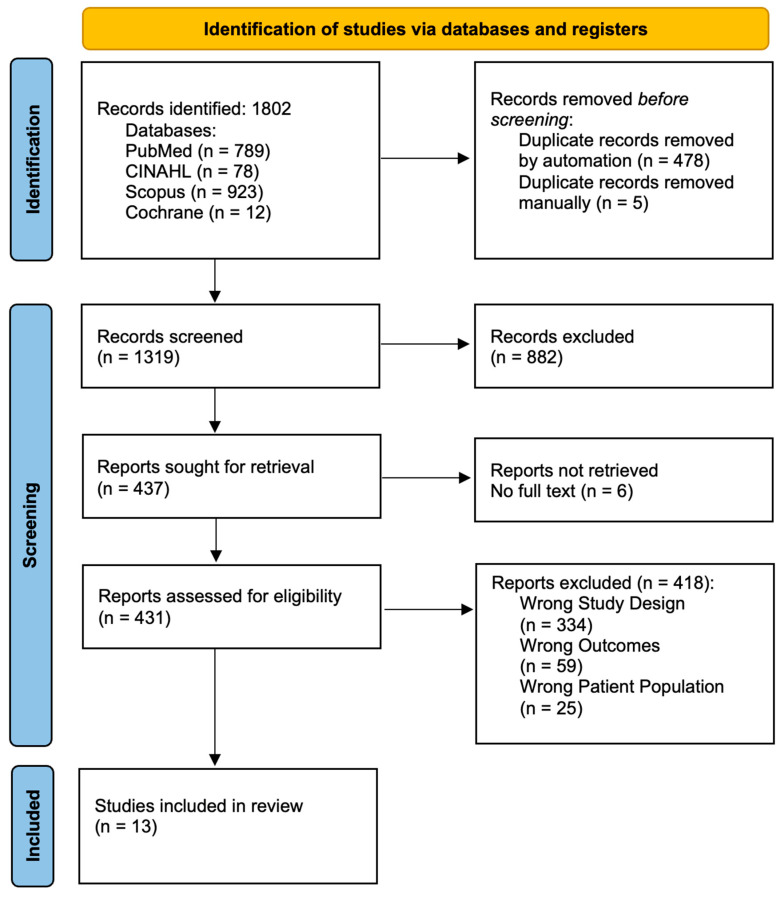
PRISMA Flow Diagram. Copyright statement: this PRISMA diagram contains public sector information licensed under the Open Government License v3.0. Adapted from: Page et al. (2020) [17].

After removal of duplicates and screening of titles and abstracts, 437 full-text articles were sought for retrieval; 6 could not be retrieved, and the remaining 431 were assessed for eligibility, of which 13 studies met inclusion criteria and were included in the systematic review (PRISMA diagram; Figure 2). All 13 studies evaluated ML models for predicting occult cervical lymph node metastasis in adults with cN0 OCSCC using pathologic nodal status as the reference standard.

### 3.2. Study Characteristics

All included studies were retrospective and were published between 2019 and 2025. Studies originated from the United States, China, Japan, Italy, Switzerland, and Australia, and most were single-center, although several used multi-institutional cohorts or registry data (Table 1).

#### Machine Learning Model Characteristics

Included studies evaluated a heterogeneous set of ML architectures and input modalities. Model types included random forests, support vector machines, extreme gradient boosting, ensemble stacking methods, artificial neural networks, LASSO, naïve Bayes, and decision tree-based methods, and penalized regression models. Input features spanned four broad domains: clinical variables alone, histopathologic features, radiomic signatures derived from preoperative imaging, and multimodal combinations of clinical and radiomic or pathologic data. One study developed and evaluated ML classifiers alongside a penalized linear nomogram that outperformed them; it met the index-test criterion of developing a ML model and contributed to the qualitative and internal-validation summary only, not the external HSROC synthesis [29].

Training datasets ranged from single-institution cohorts to multi-institutional series and one national registry-based study. Despite this diversity, external validation was reported in only 5 of 13 studies (38%), yielding 6 distinct external validation cohorts suitable for quantitative synthesis. The remaining 8 studies reported internal validation or test-set performance only and were summarized qualitatively. One externally validated cohort derived its model from the National Cancer Database (NCDB) and validated it at a single U.S. institution [19]. Given that the NCDB captures a large share of U.S. cases, patient-level overlap cannot be entirely excluded.

Among the five externally validated studies, all used tree-based or ensemble architectures, and models integrating clinical with radiomic or pathologic features tended to achieve more balanced external sensitivity and specificity than the single clinical-only model. The small number and heterogeneity of external cohorts preclude formal comparison of model types.

### 3.3. Risk of Bias and Study Quality

Risk of bias assessment using QUADAS-2 is summarized in Table 2. Risk of bias in patient selection was frequently rated high or unclear, mainly due to retrospective designs, incomplete reporting of exclusion criteria, and restriction to surgically treated patients. Risk of bias in the index test and reference standard domains was generally low, whereas the flow and timing domain was commonly rated high risk owing to incomplete reporting of neck dissection indications and timing relative to index test acquisition. The interval between index-test acquisition and neck dissection was not consistently reported across the included studies; this incomplete reporting was itself a principal driver of the high or unclear ratings in the flow-and-timing domain. Concerns regarding applicability were most often related to highly selected cohorts and the predominance of tongue subsites.

### 3.4. Pooled Characteristics of Included Patients

Across all studies, 4730 patients were included; mean age was 57.9 years (95% CI, 55.8–60.0), and 63.0% (95% CI, 53.3–72.2%) were male (Table 3). Tumor subsites were predominantly oral tongue, which accounted for 83.7% of the pooled population (95% CI, 67.6–94.9%), with smaller contributions from buccal mucosa and floor of mouth. Clinically early-stage disease was most common, with cT1 and cT2 tumors comprising 37.7% (95% CI, 28.5–47.3%) and 51.8% (95% CI, 44.6–59.0%) of patients, respectively. Pooled prevalence of occult nodal metastasis was 23.6% (95% CI, 15.5–33.0%; I^2^ = 97.3%), consistent with prior surgical series but indicating substantial between-study heterogeneity in case-mix. Pooled estimates for demographic and clinicopathologic characteristics should be interpreted with caution given substantial between-study heterogeneity (I^2^ range 49.0–98.5%).

### 3.5. Internal Validation Performance

Internally validated performance was high but variable (Table 4). AUC ranged from 0.71 to 0.96, sensitivity from 0.63 to 1.00, and specificity from 0.49 to 1.00, with the highest estimates concentrated in single-institution radiomic or multimodal models, including two reporting near-perfect discrimination [23,28]. Negative predictive value (NPV) was consistently high among studies reporting it (0.93 to 1.00). In the studies that also performed external validation, internal estimates were systematically higher than external estimates, consistent with optimism bias [21].

### 3.6. External Validation Performance

Five studies [13,19,20,21,22] reported external validation cohorts that met eligibility criteria for quantitative synthesis (Table 5). Across these cohorts, reported AUCs ranged from 0.81 to 0.92, sensitivities from 0.58 to 0.92, and specificities from 0.58 to 1.00. Among studies reporting predictive values, NPV remained high on external validation (0.94 to 0.98), whereas positive predictive value (PPV) was more modest (0.39 to 0.85), reflecting the moderate prevalence of occult nodal disease [13,21,22].

### 3.7. Pooled Diagnostic Accuracy (HSROC Meta-Analysis)

Bivariate random-effects meta-analysis of the six non-overlapping external validation cohorts yielded a pooled sensitivity of 0.79 (95% CI, 0.64 to 0.89) and pooled specificity of 0.84 (95% CI, 0.70 to 0.93) for ML models predicting occult nodal metastasis in cN0 OCSCC (Figure 2). The HSROC plot showed most cohorts clustering at relatively high sensitivity and low to moderate false-positive rates, with moderate between-study heterogeneity, particularly along the sensitivity axis. Two cohorts [13,19] lay outside the main confidence region, consistent with operating thresholds that prioritized sensitivity at the expense of lower specificity. These pooled estimates are based on a small number of externally validated cohorts and should be interpreted with caution.

### 3.8. Sensitivity Analyses

#### 3.8.1. Restriction to Low Risk of Bias

In a sensitivity analysis restricted to external validation cohorts from studies rated at low risk of bias in the QUADAS-2 patient selection domain (n = 3 cohorts), pooled sensitivity was 0.78 (95% CI, 0.60–0.89) and specificity was 0.86 (95% CI, 0.72–0.94). These estimates were similar to the primary analysis, suggesting that the summary performance was not driven solely by higher-risk studies, although the CIs were wider because of the smaller number of cohorts.

#### 3.8.2. Restricting to Models Using Only Preoperative Predictors

To explore the potential performance of models that could be implemented entirely preoperatively, we repeated the meta-analysis including only cohorts whose index models used preoperative clinical and/or radiomic features without postoperative pathology inputs (n = 4 cohorts). In this restricted analysis, pooled sensitivity was 0.81 (95% CI, 0.65–0.91) and specificity was 0.82 (95% CI, 0.67–0.91), closely mirroring the primary summary estimates. These findings suggest that ML models based solely on preoperative information can achieve diagnostic accuracy comparable to models incorporating postoperative variables, although the evidence base remains limited. The comparable performance of preoperative models may partly reflect overfitting to institution-specific imaging protocols and radiomic pipeline parameters rather than generalized biological signal; prospective replication across heterogeneous imaging environments will be necessary to distinguish these explanations.

#### 3.8.3. Excluding Outlying Cohorts

Because two cohorts appeared as outliers on the HSROC plot, we evaluated their influence by repeating the analysis after excluding [13,19] (n = 4 remaining cohorts). After exclusion of these outliers, pooled sensitivity was 0.77 (95% CI, 0.60–0.88) and specificity was 0.86 (95% CI, 0.72–0.94), with a small leftward shift in the summary point and narrower prediction regions. The consistency of summary estimates across these sensitivity analyses supports the primary HSROC findings, while underscoring the imprecision associated with the small number of externally validated cohorts.

## 4. Discussion

This DTA systematic review and meta-analysis synthesized ML models for predicting occult nodal metastasis in cN0 OCSCC, using non-overlapping external validation cohorts to estimate pooled performance. We found that external validation remains uncommon, externally validated models show promising rule-out performance with high NPV, and no study has directly compared ML-based risk stratification against depth-of-invasion (DOI)-based decision rules within the same population.

External validation was reported in only 5 of 13 included studies, yielding 6 cohorts for quantitative synthesis. The remaining studies reported internal or test-set performance only, which overestimates diagnostic accuracy through overfitting and optimism bias. Where both were reported, internally reported sensitivities and AUCs consistently exceeded external estimates, including one model whose sensitivity decreased from 1.00 internally to 0.80 on external validation [21]. High internal discrimination therefore should not be read as evidence of clinical readiness for ML-based tools in cN0 OCSCC.

Among externally validated models, pooled diagnostic performance was encouraging but not yet sufficient to justify changing standard management of the cN0 neck. The hierarchical summary receiver operating characteristic (HSROC) meta-analysis showed a pooled sensitivity of 0.79 (95% CI, 0.64–0.89) and specificity of 0.84 (95% CI, 0.70–0.93), indicating robust discrimination when models were evaluated on independent cohorts. Externally validated models also demonstrated consistently high NPV (0.94–0.98) and more modest PPV (0.39–0.85), patterns that reflect the moderate prevalence of occult nodal disease and suggest that ML models are better suited as rule-out than rule-in tools. In principle, such models could help identify a subset of cN0 patients at sufficiently low risk of occult metastasis to consider observation instead of END, but this application remains hypothetical pending prospective evaluation.

Our sensitivity analyses support the robustness of the primary HSROC findings while highlighting the limited size and heterogeneity of the evidence base. Restricting the meta-analysis to external cohorts from studies at lower risk of bias in the QUADAS-2 patient selection domain yielded pooled sensitivity and specificity estimates similar to the main analysis, with wider CIs due to fewer contributing cohorts. Likewise, analyses restricted to models based solely on preoperative clinical and/or radiomic predictors produced summary estimates that closely mirrored the overall pooled sensitivity and specificity, suggesting that models relying only on information available before surgery can achieve comparable accuracy to models incorporating postoperative pathology variables. Excluding outlying cohorts on the HSROC plot resulted in only modest movement of the summary point, indicating that the main conclusions are not driven by a small number of extreme studies, although all estimates remain imprecise because of the small number of externally validated cohorts.

Importantly, none of the included studies were designed to directly compare ML-based risk stratification with DOI-based thresholds in the same cohort. Current NCCN recommendations use a DOI threshold (for example, >3 mm) as a simple, widely available surrogate for occult nodal risk, but DOI alone cannot fully capture individual variation in metastatic potential. The most plausible role for ML is to augment, rather than replace, DOI by integrating depth alongside clinical, radiomic, and histopathologic features into individualized risk estimates. Without head-to-head comparisons of ML models and DOI-based decision rules and without decision-analytic evaluations of net clinical benefit, it remains unclear whether ML adds incremental value over existing strategies for END.

Two features of the evidence base temper how these findings should be applied. First, all included studies were retrospective and were rated at high overall risk of bias on QUADAS-2, a pattern common to the clinical prediction-model literature, so under the GRADE framework, the certainty of the pooled estimates is low and they are best read as evidence of feasibility rather than as definitive performance benchmarks. Second, because pathologic nodal status can be ascertained only in patients who undergo neck dissection, every cohort is necessarily limited to surgically managed necks. This partial-verification bias is intrinsic to the clinical question and affects essentially all diagnostic studies of occult nodal disease; its likely effect is to make sensitivity and NPV somewhat optimistic relative to an unselected cN0 population that also includes patients managed by observation. The estimates reported here should therefore be interpreted as best-case performance within surgically managed cohorts, and prospective validation in cohorts that also capture observed necks is the key next step before these models inform decisions to forgo END. Even so, the consistently high NPV reproduced across independent external cohorts is a meaningful signal that these models warrant such validation.

This review has limitations that should be considered when interpreting the findings. First, the HSROC meta-analysis is based on only six non-overlapping external validation cohorts, resulting in relatively wide CIs and limiting the precision of summary estimates. With only three to four cohorts in the sensitivity analyses, the variance components are imprecisely estimated, and the summary operating point should be read as an exploratory central estimate rather than a precise population value. Second, between-study heterogeneity was substantial, particularly in patient selection, tumor subsites, and model inputs; most cohorts focused on surgically treated tongue cancers, which may limit generalizability to the broader cN0 OCSCC population and to non-surgical management contexts. Oral tongue accounted for roughly 84% of the pooled population, so the estimates apply principally to tongue cancer. Buccal mucosa, floor of mouth, alveolar ridge, and retromolar subsites carry different rates and patterns of occult metastasis and were too sparsely represented for subsite-specific estimates. Although included cohorts spanned cTis–cT4, the great majority of patients had cT1–2 disease; the conclusions therefore apply principally to the cT1–2 population in which the elective-neck-dissection controversy is centered and should not be extended to higher T-stage disease. Because NPV is prevalence-dependent, the reported external values (0.94–0.98) were obtained in surgically managed cohorts at a pooled occult-node prevalence of 23.6%; NPV would be lower still in higher-prevalence subgroups, and because these surgical cohorts are already an enriched population, these values should not be assumed to transport directly to the lower-prevalence, observation-eligible patients in whom such a test would ultimately be applied. Third, risk of bias in patient selection and flow/timing domains was often high or unclear, reflecting retrospective designs, incomplete reporting of inclusion criteria, and variable documentation of neck dissection indications and timing, which may enrich for higher-risk disease and inflate performance estimates. Fourth, publication bias and selective reporting of models or thresholds could not be formally assessed given the small number of eligible external validation cohorts. Finally, many models relied on complex or institution-specific radiomic pipelines without standardized feature extraction or calibration procedures, which may limit reproducibility and hinder implementation in routine clinical practice.

This study also has strengths that align with current recommendations for diagnostic accuracy evidence synthesis. We addressed a clearly defined clinical question focused on occult nodal metastasis prediction in cN0 OCSCC, required pathologic nodal assessment as the reference standard, and distinguished internal from external validation, restricting quantitative synthesis to non-overlapping external cohorts to minimize optimism bias. We used QUADAS-2 to systematically evaluate risk of bias and applicability, reconstructed 2 × 2 contingency tables when necessary, and applied a bivariate random-effects HSROC model with prespecified sensitivity analyses to explore the influence of risk of bias, predictor domain, and outlying cohorts on pooled estimates.

ML-based prediction of occult nodal metastasis in cN0 OCSCC carries distinct implications for practice and research. Clinically, these models are not yet ready to guide neck management, and at present they should not influence decisions about END outside of a clinical trial. From a research perspective, the main barrier to clinical translation is no longer whether ML models can achieve favorable performance in development cohorts, but whether that performance is reproducible, calibrated, and clinically meaningful in independent populations. Most studies report apparent, internally validated, or cross-validated accuracy, metrics that may overstate real-world performance. As a result, even strong reported discrimination cannot be assumed to generalize. Future studies developing or validating ML models for this purpose should therefore treat external validation as a methodological requirement rather than an optional extension, ideally using prospective, multi-institutional cohorts with standardized imaging and pathology workflows, explicit reporting of neck management pathways, calibration assessment, and head-to-head comparison against DOI-based thresholds within the same populations. As that evidence accumulates, ML tools may ultimately help surgeons and multidisciplinary tumor boards individualize care for patients with cN0 OCSCC, but that threshold has not yet been met.

## 5. Conclusions

In this DTA systematic review and meta-analysis, ML models for predicting occult nodal metastasis in cN0 OCSCC demonstrated promising performance on non-overlapping external validation cohorts, with pooled sensitivity and specificity in ranges with potential clinical utility. Although this performance is likely an upper bound because the reference standard was verified only in surgically managed necks, consistently high NPVs suggest that such models may eventually serve as rule-out tools to identify patients at low risk of occult metastasis who could safely forgo END, whereas more modest PPVs limit their use as stand-alone rule-in tests. However, most published models lack external validation, existing external cohorts are few and heterogeneous, and no study has directly compared ML-based risk stratification with depth-of-invasion-based decision rules within the same population, so current evidence is insufficient to support routine clinical implementation. Future research should focus on prospective, multi-institutional external validation, standardized radiomic and modeling pipelines, and head-to-head and decision-analytic evaluations that test whether integrating ML into neck management pathways improves outcomes and reduces overtreatment for patients with cN0 OCSCC.

## Figures and Tables

**Table 1:** Characteristics of Machine Learning Prediction Model Studies in Patients with cN0 OCSCC.

Study	Country	Study Design	Included cT-Stage	Model Type	Model Inputs	Training Dataset	External Validation Dataset
Bur (2019) [19]	USA	Retrospective	cT1–T2	Decision forest	Clinical	NCDB	Non-Overlapping Single institution
Committeri (2022) [23]	Italy	Retrospective	cT1–T2	CART and CIDT	Multimodal (clinical + radiomics)	Multi-institutional (3)	-
Farrokhian (2022) [13]	USA	Retrospective	cT1–T2	XGBoost	Multimodal (clinical + pathologic)	Multi-institutional (7)	Non-Overlapping Single institution
Han (2024) [24]	China	Retrospective	cT1–T2	Ensemble (stacked)	Multimodal (clinical + radiomic)	Multi-institutional (2)	-
Kawamura (2023) [25]	Japan	Retrospective	cT1–4	MNN	Pathologic	Single institution	-
Kubo (2022) [26]	Japan	Retrospective	cTis–3	Random forest	Radiomic	Single institution	-
Kudoh (2023) [27]	Japan	Retrospective	cT1–4	LASSO	Radiomic	Single institution	-
Li (2025) [20]	China	Retrospective	cT1–3	Ensemble (stacked)	Multimodal (clinical + radiomic)	Multi-institutional (3)	Non-Overlapping Multi-institutional (2 cohorts, 3 centers)
Mermod (2020) [21]	Switzerland + Australia	Retrospective	cT1–4	RF	Histopathologic	Single institution	Non-Overlapping Single institution
Shan (2020) [28]	China	Retrospective	cT1–2	SVM	Multimodal (clinical + pathologic)	Single institution	-
Sun (2025) [29]	China	Retrospective	cT1–2	Linear *	Multimodal (clinical + pathologic)	Multi-institutional (2)	-
Yuan (2021) [30]	China	Retrospective	cT1–2	Naïve Bayes	Radiomic	Single institution	-
Zhu (2025) [22]	China	Retrospective	cT1–2	RF	Multimodal (clinical + radiomic)	Multi-institutional (2)	Non-Overlapping Single Institution

NCDB: national cancer database; CART: classification and regression tree; XGBoost: extreme gradient boosting; CIDT: conditional inference decision trees; MNN: multilayer perceptron neural network; LASSO: least absolute shrinkage and selection operator; RF: random forest; SVM: support vector machine. * Linear model was compared to ML models and was chosen as the more effective modality.

**Table 2:** QUADAS-2 Risk of Bias Assessment.

Study ID	Domain 1: Patient Selection			Domain 2: Index Test		Domain 3: Reference Standard		Domain 4: Flow & Timing				Domain 1: ROB	Domain 1: Concern	Domain 2: ROB	Domain 2: Concern	Domain 3: ROB	Domain 3: Concern	Domain 4: ROB	Overall ROB
	1	2	3	1	2	1	2	1	2	3	4								
Bur (2019) [19]	N	Y	U	U	N	Y	U	Y	Y	Y	Y	H	H	H	L	L	L	L	H
Committeri (2022) [23]	U	Y	Y	U	N	Y	U	Y	Y	Y	Y	U	L	H	H	L	L	L	H
Farrokhian (2022) [13]	N	Y	Y	Y	N	Y	Y	Y	Y	N	Y	H	H	H	L	L	L	U	H
Han (2024) [24]	U	Y	Y	U	N	Y	U	Y	Y	Y	Y	U	H	H	H	L	L	H	H
Kawamura (2023) [25]	U	Y	U	Y	Y	N	Y	U	N	N	N	H	H	L	H	H	H	H	H
Kubo (2022) [26]	U	Y	Y	U	N	Y	U	U	N	N	N	U	H	U	H	L	U	H	H
Kudoh (2023) [27]	U	Y	U	U	N	Y	U	Y	N	N	Y	H	H	H	H	U	L	H	H
Li (2025) [20]	U	Y	Y	Y	N	Y	U	Y	Y	Y	Y	U	H	H	H	L	L	L	H
Mermod (2020) [21]	U	Y	U	U	N	Y	U	Y	Y	Y	Y	H	H	H	L	L	L	L	H
Shan (2020) [28]	U	Y	Y	U	N	Y	U	Y	Y	Y	Y	U	L	H	H	L	L	L	H
Sun (2025) [29]	U	Y	Y	Y	N	Y	U	Y	Y	Y	Y	U	H	H	H	L	L	L	H
Yuan (2021) [30]	U	Y	Y	U	N	Y	U	Y	Y	Y	Y	U	H	H	H	L	L	L	H
Zhu (2025) [22]	U	Y	Y	U	N	Y	U	Y	Y	Y	Y	U	H	H	H	L	L	L	H

ROB: risk of bias. Signaling-question responses: Y, yes; N, no; U, unclear. Domain-level ratings: L, low; H, high; U, unclear. Numbered columns within each domain correspond to the QUADAS-2 signaling questions; the ROB and Concern columns give the domain-level summary judgment.

**Table 3:** Pooled Demographic and Clinicopathologic Characteristics of Included Patients.

	Observed [Raw]	Overall [Pooled]	95% CI	I^2^
**Patients, n**	4730	N/A	N/A	N/A
**Age, mean (years)**	4730	57.9	(55.8 to 60.0)	N/A
Range (years)	318	19–92	N/A	N/A
**Sex**				
Male	1681/2698	63.0%	(53.3% to 72.2%)	95.9%
Female	1017/2698	37.0%	(27.8% to 46.7%)	95.9%
**Subsite**				
Alveolar Ridge	178/2747	4.0%	(1.5% to 7.7%)	89.9%
Buccal	401/2997	-	-	-
Floor of Mouth	363/2747	8.1%	(3.4% to 14.5%)	94.1%
Lip	103/2747	1.5%	(0.0% to 5.9%)	95.9%
Palate	37/2747	1.4%	(0.6% to 2.5%)	60.0%
Retromolar Trigone	20/2747	-	-	-
Tongue	2014/2964	83.7%	(67.6% to 94.9%)	98.5%
Other	51/2747	1.9%	(1.4% to 2.4%)	49.0%
**cT Stage**				
cTis	28/161	-	-	-
cT1	2152/4015	37.7%	(28.5% to 47.3%)	96.6%
cT2	1751/4015	51.8%	(44.6% to 59.0%)	93.8%
cT3	48/713	8.5%	(3.7% to 15.2%)	86.0%
cT4	36/284	14.5%	(7.9% to 22.6%)	63.7%
**Occult Node Status**				
Positive	868/4256	23.6%	(15.5% to 33.0%)	97.3%

Observed [Raw]: count and denominator across studies reporting each characteristic. Overall [Pooled]: random-effects pooled estimate. Pooled and raw proportions may differ because random-effects pooling weights studies approximately equally and is calculated over the subset of studies reporting each variable; pooled categories are not constrained to sum to 100%. Buccal subsite pooled estimate suppressed as the 95% CI spans nearly the entire range and is therefore uninterpretable.

**Table 4:** Internal Validation or Test Set Performance.

	Sensitivity (95% CI)	Specificity (95% CI)	Accuracy	AUC (95% CI)	PPV (95% CI)	NPV (95% CI)
Bur (2019) [19]	0.75	0.49	-	0.71	-	-
Committeri (2022) [23]	1.00	1.00	1.00	-	-	-
Farrokhian (2022) [13]	0.94	0.78	0.78	0.90	0.49	0.98
Han (2024) [24]	1.00	0.77	0.84	0.95	-	-
Kawamura (2023) [25]	-	-	0.99	-	-	-
Kubo (2022) [26]	0.82	-	0.85	0.92	-	-
Kudoh (2023) [27]	0.65	0.70	0.68	0.79	-	-
Li (2025) [20]	0.67	0.89	0.77	0.85		
Mermod (2020) [21]	1.00 (0.90 to 1.00)	0.98 (0.90 to 1.00)	-	0.89 (0.8 to 0.98)	0.98 (0.90 to 1.00)	1.00 (0.93 to 1.00)
Shan (2020) [28]	1.00	0.88	-	0.96	-	-
Sun (2025) [29]	-	-	-	0.78	0.21	0.97
Yuan (2021) [30]	0.63	0.82	0.74	0.80	-	-
Zhu (2025) [22]	0.90	0.93	-	0.94 (0.91 to 0.98)	0.87	0.93

**Table 5:** External Model Performance.

	Sensitivity (95% CI)	Specificity (95% CI)	Accuracy (95% CI)	AUC (95% CI)	PPV (95% CI)	NPV (95% CI)
Bur (2019) [19]	0.92	0.58	-	0.84	-	-
Farrokhian (2022) [13]	0.92	0.73	-	0.84	0.39	0.98
Li (2025) [20] #1	0.62	0.87	0.76	0.81	-	-
Li (2025) [20] #2	0.58	1.00	0.89	0.82	-	-
Mermod (2020) [21]	0.80 (0.60 to 0.90)	0.90 (0.80 to 0.90)	0.90 (0.80 to 0.90)	0.85 (0.80 to 0.90)	0.60 (0.40 to 0.80)	0.95 (0.90 to 1.00)
Zhu (2025) [22]	0.87	0.91	-	0.92 (0.87 to 0.97)	0.81	0.94

## Data Availability

This study is a systematic review and meta-analysis of previously published data. All data supporting the findings are publicly accessible within the included primary studies and their Supplementary Materials, which are cited in the reference list. The datasets generated during the review—including the extracted diagnostic-accuracy estimates and the reconstructed 2 × 2 contingency-table data used in the meta-analysis—are provided in Supplementary Tables S1 and S2. No new primary data were generated. Further details are available from the corresponding author upon reasonable request.

## Supplementary Materials

The following supporting information can be downloaded at https://www.mdpi.com/article/10.3390/cancers18142271/s1. Table S1. Reconstructed 2 × 2 contingency-table data. Table S2. Univariate cross-check of pooled sensitivity and specificity. Table S3. Full search strategy.

## Abbreviations

The following abbreviations are used in this manuscript:

OCSCC	Oral Cavity Squamous Cell Carcinoma
cN0	Clinically Node Negative
END	Elective Neck Dissection
ML	Machine Learning
DOI	Depth of Invasion
AUC	Area Under the Curve
HSROC	Hierarchical Summary Receiver Operating Characteristic
QUADAS-2	Quality Assessment of Diagnostic Accuracy Studies–Version 2
NPV	Negative Predictive Value
PPV	Positive Predictive Value
NCCN	National Comprehensive Cancer Network
PRISMA	Preferred Reporting Items for Systematic Reviews and Meta-Analyses
CI	Confidence Interval
