# Supplementary material for: Performance of Machine Learning Models for Predicting Occult Nodal Metastasis in Oral Cavity Squamous Cell Carcinoma: A Systematic Review and Diagnostic Accuracy Meta-Analysis
Source: Cancers (Basel). 2026 Jul 15;18(14):2271. doi: 10.3390/cancers18142271 (PMC13406554; doi:10.3390/cancers18142271)
Supplement: Supplementary file 1 [file cancers-18-02271-s001.zip › cancers-4380940-supplementary.pdf]

Supplementary Table S1. Reconstructed 2×2 contingency-table data

		Reference Standard		2×2 Table					
	N	*Positive*	*Negative*	TP	FP	FN	TN	Sensitivity	Specificity
**Bur**	71	12	59	11	25	1	34	0.92	0.58
**Farrokhian**	148	24	124	22	34	2	90	0.92	0.73
**Li #1**	63	28	35	17	5	11	30	0.62	0.87
**Li #2**	45	12	33	7	0	5	33	0.58	1.00
**Mermod**	112	20	92	16	9	4	83	0.80	0.90
**Zhu**	124	38	86	33	8	5	78	0.87	0.91

TP = true positive; FP = false positive; FN = false negative; TN = true negative.

Supplementary Table S2. Univariate cross-check of pooled sensitivity and specificity

	Cohorts	*Pooled Estimate*	*95% CI*	I^2	τ^2
**Sensitivity**	6	0.80	0.67 to 0.89	53.3	0.368
**Specificity**	6	0.86	0.72 to 0.94	89.9	1.031

Supplementary Table S3. Full search strategy.

Database	Search Strategy
**PubMed**	("Squamous Cell Carcinoma of Head and Neck"[Mesh] OR "oral cavity squamous cell carcinoma" OR OCSCC OR "tongue squamous cell carcinoma" OR "mouth neoplas*" OR "oral cancer" OR "oral cavity cancer" OR "oral tongue carcinoma" OR "oral squamous cell carcinoma" OR "oral tongue squamous cell carcinoma" OR "floor of mouth cancer" OR "floor of mouth carcinoma" OR "mouth cancer" OR "buccal cancer" OR "tongue cancer" OR "buccal carcinoma" OR "mandibular cancer" OR "mandibular carcinoma" OR "alveolar ridge cancer" OR "alveolar ridge carcinoma" OR "retromolar trigone cancer" OR "retromolar trigone carcinoma" OR "hard palate cancer" OR "hard palate carcinoma") AND ("Machine Learning"[Mesh] OR "machine learning" OR "machine-learning" OR "deep learning" OR "deep-learning" OR "supervised learning" OR "unsupervised learning" OR "neural network*" OR "convolutional neural network*" OR "support vector machine*" OR "radiomic*" OR "feature selection" OR "LASSO" OR "penalized" OR "least absolute shrinkage" OR "survival prediction" OR "prediction model") AND (Outcomes OR OS OR "overall survival" OR "overall-survival" OR

Database	Search Strategy
	EFS OR "event free survival" OR "event-free survival" OR DFS OR "disease free survival" OR "disease-free survival" OR PFS OR "progression free survival" OR "progression-free survival" OR TTP OR "time to progression" OR "time-to-progression" OR "time-to-event" OR recurren* OR RFS OR "recurrence free survival" OR "recurrence-free survival" OR "locoregional recurrence" OR mortality OR death OR "nodal metastasis" OR "occult lymph node metastasis" OR "occult lymph node metastases")
**Scopus**	TITLE-ABS-KEY("oral cavity squamous cell carcinoma" OR OCSCC OR "tongue squamous cell carcinoma" OR "mouth neoplas*" OR "oral cancer" OR "oral cavity cancer" OR "oral squamous cell carcinoma" OR "oral tongue carcinoma" OR "oral tongue squamous cell carcinoma" OR "floor of mouth cancer" OR "floor of mouth carcinoma" OR "mouth cancer" OR "buccal cancer" OR "buccal carcinoma" OR "tongue cancer" OR "mandibular cancer" OR "mandibular carcinoma" OR "alveolar ridge cancer" OR "alveolar ridge carcinoma" OR "retromolar trigone cancer" OR "retromolar trigone carcinoma" OR "hard palate cancer" OR "hard palate carcinoma") AND TITLE-ABS-KEY("machine learning" OR "machine-learning" OR "deep learning" OR "deep-learning" OR "supervised learning" OR "unsupervised learning" OR "neural network*" OR "convolutional neural network*" OR "support vector machine*" OR radiomic* OR "feature selection" OR LASSO OR penalized OR "least absolute shrinkage" OR "survival prediction" OR "prediction model") AND TITLE-ABS-KEY(Outcomes OR OS OR "overall survival" OR "overall-survival" OR EFS OR "event free survival" OR "event-free survival" OR DFS OR "disease free survival" OR "disease-free survival" OR PFS OR "progression free survival" OR "progression-free survival" OR TTP OR "time to progression" OR "time-to-progression" OR "time-to-event" OR recurren* OR RFS OR "recurrence free survival" OR "recurrence-free survival" OR "locoregional recurrence" OR mortality OR death OR "nodal metastasis" OR "occult lymph node metastasis" OR "occult lymph node metastases")
**CINAHL**	[Title, Abstract] ("oral cavity squamous cell carcinoma" OR OCSCC OR "tongue squamous cell carcinoma" OR "mouth neoplas*" OR "oral cancer" OR "oral cavity cancer" OR "oral squamous cell carcinoma" OR "oral tongue carcinoma" OR "oral tongue squamous cell carcinoma" OR "floor of mouth cancer" OR "floor of mouth carcinoma" OR "mouth cancer" OR "buccal cancer" OR "buccal carcinoma" OR "tongue cancer" OR "mandibular cancer" OR "mandibular carcinoma" OR "alveolar ridge cancer" OR "alveolar ridge carcinoma" OR "retromolar trigone cancer" OR "retromolar trigone carcinoma" OR "hard palate cancer" OR "hard palate carcinoma") AND ("machine learning" OR "machine-learning" OR "deep learning" OR "deep-learning" OR "supervised learning" OR "unsupervised learning" OR "neural network*" OR "convolutional neural network*" OR "support vector machine*" OR radiomic* OR "feature selection" OR LASSO OR penalized OR "least absolute shrinkage" OR "survival prediction" OR "prediction model") AND (Outcomes OR OS OR "overall survival" OR "overall-survival" OR EFS OR "event free survival" OR "event-free survival" OR DFS OR "disease free survival" OR

Database	Search Strategy
	"disease-free survival" OR PFS OR "progression free survival" OR "progression-free survival" OR TTP OR "time to progression" OR "time-to-progression" OR "time-to-event" OR recurren* OR RFS OR "recurrence free survival" OR "recurrence-free survival" OR "locoregional recurrence" OR mortality OR death OR "nodal metastasis" OR "occult lymph node metastasis" OR "occult lymph node metastases")
**Cochrane**	[Title, Abstract, Keyword] ("oral cavity squamous cell carcinoma" OR OCSCC OR "tongue squamous cell carcinoma" OR "mouth neoplas*" OR "oral cancer" OR "oral cavity cancer" OR "oral squamous cell carcinoma" OR "oral tongue carcinoma" OR "oral tongue squamous cell carcinoma" OR "floor of mouth cancer" OR "floor of mouth carcinoma" OR "mouth cancer" OR "buccal cancer" OR "buccal carcinoma" OR "tongue cancer" OR "mandibular cancer" OR "mandibular carcinoma" OR "alveolar ridge cancer" OR "alveolar ridge carcinoma" OR "retromolar trigone cancer" OR "retromolar trigone carcinoma" OR "hard palate cancer" OR "hard palate carcinoma") AND ("machine learning" OR "machine-learning" OR "deep learning" OR "deep-learning" OR "supervised learning" OR "unsupervised learning" OR "neural network*" OR "convolutional neural network*" OR "support vector machine*" OR radiomic* OR "feature selection" OR LASSO OR penalized OR "least absolute shrinkage" OR "survival prediction" OR "prediction model") AND (Outcomes OR OS OR "overall survival" OR "overall-survival" OR EFS OR "event free survival" OR "event-free survival" OR DFS OR "disease free survival" OR "disease-free survival" OR PFS OR "progression free survival" OR "progression-free survival" OR TTP OR "time to progression" OR "time-to-progression" OR "time-to-event" OR recurren* OR RFS OR "recurrence free survival" OR "recurrence-free survival" OR "locoregional recurrence" OR mortality OR death OR "nodal metastasis" OR "occult lymph node metastasis" OR "occult lymph node metastases")
